# Linking Murine and Human Plasmodium falciparum Challenge Models in a Translational Path for Antimalarial Drug Development

**DOI:** 10.1128/AAC.02883-15

**Published:** 2016-05-23

**Authors:** James S. McCarthy, Louise Marquart, Silvana Sekuloski, Katharine Trenholme, Suzanne Elliott, Paul Griffin, Rebecca Rockett, Peter O'Rourke, Theo Sloots, Iñigo Angulo-Barturen, Santiago Ferrer, María Belén Jiménez-Díaz, María-Santos Martínez, Rob Hooft van Huijsduijnen, Stephan Duparc, Didier Leroy, Timothy N. C. Wells, Mark Baker, Jörg J. Möhrle

**Affiliations:** a QIMR Berghofer Medical Research Institute, Brisbane, Australia; b University of Queensland, Brisbane, Australia; c QPharm Pty. Ltd., Brisbane, Australia; d Mater Health Services, Brisbane, Australia; e Queensland Paediatric Infectious Diseases (QPID), Herston, Australia; f GlaxoSmithKline, Tres Cantos Drug Development Campus, Diseases of the Developing World, Tres Cantos, Spain; g Medicines for Malaria Venture, Geneva, Switzerland

## Abstract

Effective progression of candidate antimalarials is dependent on optimal dosing in clinical studies, which is determined by a sound understanding of pharmacokinetics and pharmacodynamics (PK/PD). Recently, two important translational models for antimalarials have been developed: the NOD/SCID/IL2Rγ^−/−^ (NSG) model, whereby mice are engrafted with noninfected and Plasmodium falciparum-infected human erythrocytes, and the induced blood-stage malaria (IBSM) model in human volunteers. The antimalarial mefloquine was used to directly measure the PK/PD in both models, which were compared to previously published trial data for malaria patients. The clinical part was a single-center, controlled study using a blood-stage Plasmodium falciparum challenge inoculum in volunteers to characterize the effectiveness of mefloquine against early malaria. The study was conducted in three cohorts (*n =* 8 each) using different doses of mefloquine. The characteristic delay in onset of action of about 24 h was seen in both NSG and IBSM systems. *In vivo* 50% inhibitory concentrations (IC_50_s) were estimated at 2.0 μg/ml and 1.8 μg/ml in the NSG and IBSM models, respectively, aligning with 1.8 μg/ml reported previously for patients. In the IBSM model, the parasite reduction ratios were 157 and 195 for the 10- and 15-mg/kg doses, within the range of previously reported clinical data for patients but significantly lower than observed in the mouse model. Linking mouse and human challenge models to clinical trial data can accelerate the accrual of critical data on antimalarial drug activity. Such data can guide large clinical trials required for development of urgently needed novel antimalarial combinations. (This trial was registered at the Australian New Zealand Clinical Trials Registry [http://anzctr.org.au] under registration number ACTRN12612000323820.)

## INTRODUCTION

The cumulative success rate from candidate selection to registration for the development of anti-infectives is below 5% (benchmark data from the Centres for Medicines Research [2008 to 2011] as cited in reference 1). There are multiple reasons for this low success rate, but a recent survey cites “uncertainties related to dose selection” as the top cause for unsuccessful FDA applications (2). One of the key challenges in moving drugs from animal studies into the clinic, where there is very limited opportunity for testing different regimens, is to provide reasonable prior estimates to inform the design of clinical studies that will have optimal safety and efficacy in patients.

Major technical improvements have been made in the past few years in preclinical development of antimalarial drugs. These improvements have included improved rodent models (3–6) that better replicate human malaria, more comprehensive pharmacometric modeling (7, 8), and better design of proof-of-concept clinical trials (9, 10). Drawbacks of the older “standard” mouse model for malaria include the use of a rodent parasite, Plasmodium berghei, with different growth kinetics and drug response from those of the major human pathogen Plasmodium falciparum and the fact that the parasite resides within murine, instead of human, erythrocytes. Moreover, the older standard preclinical assay known as Peter's test (11) is not commensurable with clinical studies because its design only allows assessing how parasite growth is delayed by drug treatment and not the parasite killing or clearance under therapeutic treatment (12). The development of the NOD SCID IL-2Rγ^−/−^ (NSG) immunodeficient rodent model of malaria (P. falciparum humanized mouse model, described in reference 13) represents an improved standard for preclinical *in vivo* studies of blood-stage antimalarial drugs. In this model, NSG mice engrafted with human erythrocytes are susceptible to infection by the human pathogen P. falciparum, which allows a precise analysis of the parasitological response to different exposure levels of drugs *in vivo* (14). Importantly, the experimental design used in preclinical studies allows production of estimates for all pharmacodynamic (PD) data of interest obtained in clinical trials assessing both parasite clearance and recrudescence.

A reliably predictive transition from mice to humans remains crucial and must overcome considerable differences between murine and human pharmacokinetics (PK) and their overall response to malaria (15). One approach to obtaining more rapid proof of concept in humans is the controlled human malaria infection protocol. Volunteers can be infected following the bite of infected mosquitoes (reviewed in reference 10) or by intravenous (i.v.) injection with cryopreserved sporozoites (16). Such sporozoite-induced malaria is particularly well suited to test vaccines or drugs that act prior to the development of blood-stage malaria, i.e., directly against sporozoites or targeting infected hepatocytes. More recently, an experimentally induced blood-stage malaria (IBSM) model has been developed as a system to test antimalarial drugs with activity against the blood stages of the parasite (9, 17), using parasite-infected human erythrocytes. By closely monitoring parasite multiplication using quantitative PCR (qPCR) (18), rich pharmacodynamic data can be obtained over an effective dynamic range of >1,000-fold at levels of parasitemia several orders of magnitude below those causing clinical malaria. Moreover, time to recrudescence (for up to 4 weeks) can be measured.

To investigate the potential of the combined use of these two models, we evaluated a well-established and metabolically stable malaria drug, mefloquine (19), in both and compared our findings with earlier observations regarding mefloquine in more traditional phase II clinical trials with malaria patients.

## MATERIALS AND METHODS

### Induced blood-stage infection and mefloquine treatment in NSG mice.

*In vivo* therapeutic efficacy against P. falciparum was assessed in a 4-day parasite-normalized standard assay (PNSA) previously described (12). NOD SCID IL-2Rγ^−/−^ (NSG) mice (Charles River, France) were injected daily with 1 ml of human erythrocytes (50% hematocrit) (20) and approximately 10 days later infected i.v. with 2.0 × 10^7^ erythrocytes infected with nonsynchronized P. falciparum (strain 3D7^0087/N9^) obtained from P. falciparum-infected donor mice. The Plasmodium strain had been generated at GlaxoSmithKline (GSK), Tres Cantos, Spain (20). Parasitemia was measured every 24 h from days 3 to 7 after infection.

Four *in vivo* studies were performed in the P. falciparum humanized mouse model: (i) *per os* (p.o.) multidose administration once a day for 4 days at 0.2, 1, 3, 10, and 30 mg/kg of body weight (*n =* 3 mice per dose level); (ii) multidose administration p.o. once a day for 4 days at 1, 2.5, 5, 10, 20, 30, 40, 50, 60, and 70 mg/kg (*n =* 1 mouse per dose level); (iii) bolus i.v. single administration at 1 mg/kg in both infected (P. falciparum) and noninfected humanized mice (*n =* 3 mice per group); and (iv) single administration p.o. at 20 mg/kg of infected (P. falciparum) and noninfected humanized mice (*n =* 3 mice per group). Data from the three experiments were used to model exposure kinetics.

Mefloquine levels in whole blood were measured in serial samples of peripheral blood (25 μl) from every mouse in the *in vivo* studies. The samples were taken at 0.25, 0.5, 1, 2, 3, 6, 8, and 23 h after the first dose and diluted with 25 μl of water containing 0.1% saponin for erythrocyte lysis, flash-frozen on dry ice, and stored at −80°C until analysis. The lower limit of quantification of mefloquine in blood was 2.5 ng/ml.

Parasitemia was measured in experiments i and ii by flow cytometry as described previously (21), with a detection limit of 0.01% (measuring infected erythrocytes).

### Induced blood-stage malaria clinical study.

A single-center, controlled study was undertaken by following methods previously described (9). The study was designed to be undertaken in three cohorts of eight volunteers receiving a single treatment dose of mefloquine (Lariam, Roche) at 5, 10, or 15 mg/kg. After screening conducted up to 4 weeks prior to enrollment, eligible participants were inoculated on day 0 with ∼1,800 viable P. falciparum-infected human erythrocytes administered i.v. (9, 22). Participants were monitored on an outpatient basis for adverse events and for unexpected early onset of symptoms and signs or parasitological evidence of malaria. On the day designated for commencement of treatment, as determined by qPCR (18) results or clinical manifestation of malaria, participants were admitted to the study unit and confined for 48 h for mefloquine administration, determination of parasite load and drug levels, and safety monitoring. The threshold for commencement of mefloquine treatment was 1,000 parasites/ml (by qPCR of Plasmodium 18S rRNA genes). If clinical or parasitological evidence of malaria (the onset of clinical features of malaria) occurred, treatment began at this time. If clinically well at the end of the 48 h confinement period, participants were discharged and monitored on an outpatient basis for safety, blood mefloquine levels, and the presence of malaria parasites by qPCR. The effect of mefloquine on parasitemia was observed for up to 7 days before compulsory commencement of treatment with artemether-lumefantrine (20/120 mg; Riamet; Novartis) administered as four tablets as a single dose every 12 h for 60 h. As an additional safety measure, parasitemia was monitored until the end-of-study visit, scheduled approximately 28 days after the inoculation. Dose escalation between cohorts occurred only after review by the Safety Review Team of the observed mefloquine safety and pharmacodynamic outcome up to day 14 postinoculation for the previous cohort. Details of parasitemia and plasma mefloquine determinations are described in the supplemental material.

### Measurement of parasitemia in human volunteers.

Parasitemia was quantified by qPCR as described previously (18) at the following time points: (i) pretreatment, i.e., baseline following inoculation and then twice daily from day 3 until qPCR indicated ≥1,000 parasites/ml or clinical features of malaria occurred; (ii) after mefloquine dosing at 0, 2, 4, 8, 12, 24, 36, 48, 60, 72, 84, 96, 108, 120, 132, 144, and 156 h; and (iii) on the morning of terminal curative therapy with artemether-lumefantrine, on the two subsequent mornings, and at the end-of-study visit.

### Human mefloquine exposure and PK.

The PK of mefloquine was assessed in terms of the plasma concentration-time profiles in blood samples collected predose and at the following times after dosing: 0.25, 0.5, 1, 2, 3, 4, 6, 8, 12, 24, 36, 48, 72, 96, 120, and 144 h. Plasma was separated and frozen at ≤−70°C within 30 min. It was found that mefloquine standards in plasma could be stored for 10 days at 4°C with no change in measured concentration (data not shown). Mefloquine was assayed in 200-μl plasma samples following acetonitrile extraction as described previously (20). For PK assessment, all observations below the lower limit of quantitation were reported as <25 ng/ml.

### Statistical methods. (i) PK Modeling.

One- and two-compartment PK models were evaluated, with and without a lag in first-order absorption (see supplemental material for details). A one-compartment model had been used previously to model mefloquine exposure (23). A two-compartment model provided the best fit for the NSG mice in our study, with two-compartment PK clearly demonstrated after i.v. administration (see Fig. S2 and S3 in the supplemental material). This model provided a robust characterization of the observed PK data. There was no evidence of an effect of inoculation with parasites on the mefloquine PK (data not shown).

Concentration-versus-time data were analyzed by noncompartmental analysis methods using Phoenix software (v.6.3; Pharsight). Additional statistical analysis and modeling were performed with GraphPad Prism (v.5.01; GraphPad Software Inc., San Diego, CA) and NONMEM software (v.7.2; ICON Development Solutions, MD).

### (ii) PRR calculations.

Parasite reduction ratio (PRR) estimates were calculated as previously described (24). In brief, the decay rate (slope coefficient from the log linear decay regression) for each individual was calculated, and then the weighted average slope estimate and corresponding standard error were calculated using an inverse-variance method. All parasitemia values were log_10_ transformed, and the mean of the log_10_-transformed parasitemia was implemented as a summary parasitemia value per time point per subject. For the historical data sets for which aggregated parasitemia data were available, PRR estimates were calculated using the mean parasitemia for each study and dose of interest over time (hours since treatment).

### PK/PD modeling.

The parasitemia data were modeled as part of the simultaneous PK and PD (PK/PD) modeling (25). Successful modeling of parasitemia data required inclusion of the lag and its time-dependent trend.

The PK/PD model was compiled in NONMEM using differential equations based on the initial PK and baseline modeling. Our earlier PK/PD model (25), based on reference 26, provided a good fit for the NSG mouse model as shown overlaid graphically on the data points in Fig. S2 in the supplemental material. The PD portion of the model included the first-order growth characterized with the control animals and a drug effect characterized with a first-order elimination of parasitemia with the novel lag phase function used to describe the elimination rate constant:

$$\frac{dP}{dt}=P\times \left(k_{G}-k_{D}\times \frac{C^{H}}{{\text{IC}}_{50}+C^{H}}\right)$$

where *k_G_* is a first-order parasite growth constant, *k_D_* is a first-order parasite clearance constant, *C* is the drug concentration in the effect compartment, IC_50_ is the drug concentration corresponding to when parasite clearance is half maximal, and *H* is the Hill coefficient, determining the steepness of the drug concentration-parasite clearance relationship.

### Ethical considerations.

The clinical protocol and associated documents were reviewed and approved by the Queensland Institute of Medical Research Berghofer Human Research Ethics Committee. The trial was registered at anzctr.org.au (registration number ACTRN12612000323820). All the animal experiments performed at GSK were approved by the DDW Ethical Committee on Animal Research, performed at the DDW Laboratory Animal Science facilities accredited by AAALAC, and conducted in accordance with European Directive 86/609/EEC and the GSK policy on the care, welfare, and treatment of animals (34). The human biological samples used at GSK were sourced ethically, and their research use was in accord with the terms of the informed consent.

## RESULTS

### PK/PD of mefloquine in the P. falciparum-infected NOD SCID mouse model.

**FIG 1:**
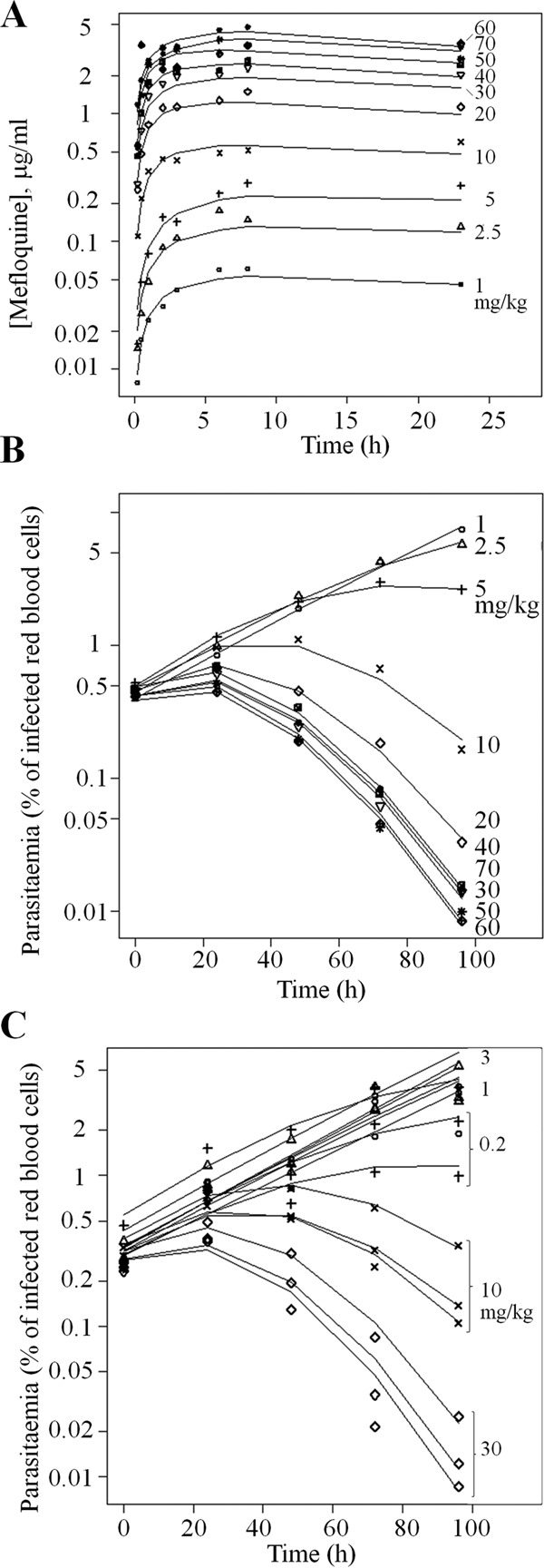
Mefloquine exposure and parasitemia in P. falciparum-infected NOD SCID IL-2Rγ^−/−^ (NSG) mice. (A) Mefloquine concentrations in blood observed over time for various oral dosings. The lines represent the PK/PD model fit using parameters listed in Tables 1 and 2. (B and C) Mefloquine efficacy in P. falciparum-infected NSG mice. (B) Oral administration once daily for 4 days at 1, 2.5, 5, 10, 20, 30, 40, 50, 60, and 70 mg/kg/day (*n =* 1). (C) Same as in panel B but with dosing at 0.2, 1, 3, 10, and 30 mg/kg (*n =* 3). Results for the 0.2-, 1-, and 3-mg/kg dosings overlap. The observed data points were overlaid with model predictions (see the text).

Mefloquine exposure (Fig. 1A) was dose dependent, with a half-life significantly longer than the 23-h duration of observation. Parasitemia increased in a log linear manner in infected control animals (see Fig. S1 in the supplemental material), with an average log_10_ growth rate of 0.013 h^−1^ (coefficient of variation [CV], 10%). In mefloquine-treated animals (Fig. 1B and C), a dose-dependent response with a lag in onset of effect was observed. When the PK (see Fig. S2) data were analyzed simultaneously with the parasite clearance data (see Fig. S3) using a nonlinear mixed-effects approach, the maximum log_10_ parasite reduction ratio (log_10_ PRR_Max_) was calculated as 3.3 (see Tables 1 and 2 for all PK/PD parameters).

**TABLE 1:** Mefloquine PK key parameters in NOD SCID IL-2Rγ^−/−^ mice

Parameter^*a*^	Estimate	Relative SE	95% confidence interval
Theta			
Ka	0.34/h	26	0.17–0.51
*V*	2,810 ml/kg	37	772–4,580
CL	162 ml/h/kg	14	117–207
*V*_2_	5,110 ml/h/kg	15	3,640–6,580
*Q*	2,730 ml/h/kg	2.8	2,580–2,880
*F*	0.56%	16	0.39–0.73
Eta			
Ka	0.28	39	
*V*	0.61	50	
CL	0.065	31	
Sigma			
PK	0.0319		

a CL, clearance; *F*, extent of absorption or bioavailability; Ka, absorption rate constant; *Q*, intercompartmental clearance; *V*, central volume; *V*_2_, peripheral volume. See supplemental material for other model parameters.

**TABLE 2:** Mefloquine PD key parameters in NOD SCID IL-2Rγ^−/−^ mice

Parameter^*a*^	Estimate	Relative SE	95% confidence interval	Derived parameter	Estimate	95% confidence interval
Theta						
*G*	0.0312	4.5	0.029–0.034	Growth rate	0.65/48 h	0.59–0.71
*D*_max_	0.189	22	0.11–0.27	Death rate	3.94/48 h	2.2–5.7
*k*	0.00892/h	30	0.0036–0.014	Log_10_ PRR_48_	3.29	
IC_50_	0.768 μg/ml	8.3	0.64–0.89			
*H*	1.81	25	0.94–2.68			
BL	0.376% parasitemia	1	0.369–0.383			
Eta						
*G*	0.15	40				
BL	0.046	26				
Sigma						
PD	0.0364					

a *D*_max_, maximal death rate; *G*, growth rate; *H*, Hill coefficient; *k*, exponential rate constant describing the acceleration of parasitemia elimination; BL, basal level. See supplemental material for other model parameters.

### Testing of mefloquine in the IBSM clinical study.

Twenty-two volunteers (11 male and 21 Caucasian) were enrolled in a clinical trial to evaluate the PK/PD relationship of mefloquine administered in three doses (5 mg/kg [*n =* 6], 10 mg/kg [*n =* 8], and 15 mg/kg [*n =* 8]). The average age of volunteers was 25 (±4) years, with a weight of 72 (±11) kg, a height of 175 (±11) cm, and body mass index (BMI) of 23 (±2). The only serious adverse event (recurrent tonsillitis) was considered unrelated to the clinical trial treatment. The mild and moderate adverse events were either malaria related or mefloquine related (*n =* 52 and *n =* 22, respectively; see Table S2 in the supplemental material). These events were comparable to those seen earlier in the IBSM studies or reported following the use of mefloquine. Among the 52 adverse events attributed to malaria, all were judged to be of mild to moderate severity, and they were generally of a transient nature. The most common adverse events attributable to malaria were fever symptoms (*n =* 6) and mild leukopenia (*n =* 7). Of the adverse events attributable to mefloquine, all except two (lucid dreams and nausea) occurred in the highest-dose cohort (15 mg/kg). Of note, three of the five subjects who experienced dizziness reported a significant duration of symptoms (4, 12, and 20 days). Four subjects reported nausea and/or vomiting following receipt of the highest dose of mefloquine, and two reported insomnia, with one of these two reporting that this lasted for 6 days.

### PK/PD of mefloquine in induced blood-stage malaria in human volunteers.

**FIG 2:**
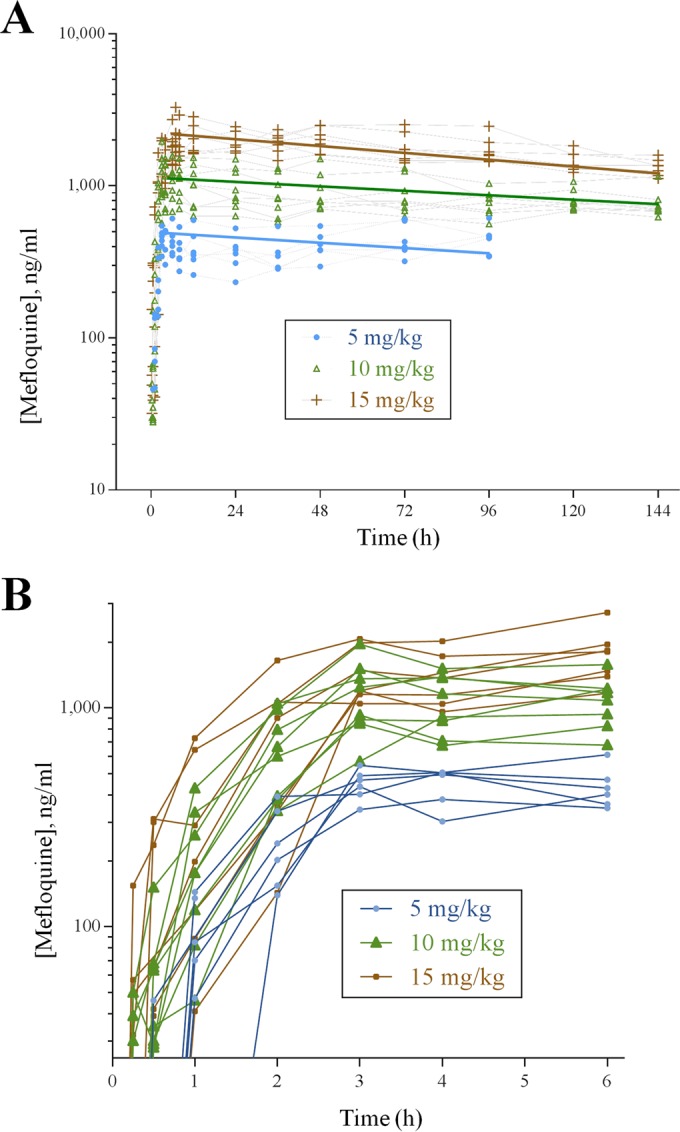
(A) Time course of individual mefloquine levels following the oral administration of 5, 10, and 15 mg/kg in volunteers, logarithmic scale, overlaid with graphs using modeled parameters for each dose. The limit of detection was 25 ng/ml. (B) Mefloquine exposure for the three cohorts in the 1- to 6-h interval.

Orally administered mefloquine in IBSM displayed exponential kinetics (Fig. 2), which were approximately linear (see Table S1). The noncompartmental PK parameters confirmed the increase with dose to be supraproportional (see Fig. S4). A one-compartment with first-order absorption, lag time, and dose-dependent volume of distribution was determined to most accurately describe the data, as shown in Fig. 2, in which the modeled data are graphically overlaid. The half-life of mefloquine derived from the PK model fit was 200 h (8.3 days).

**FIG 3:**
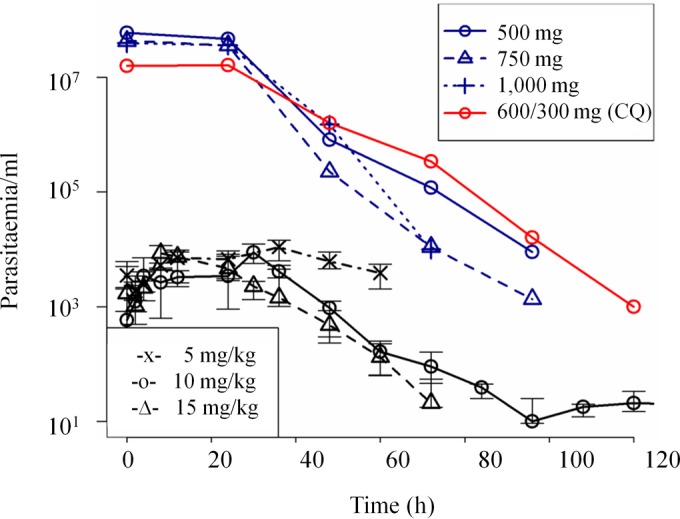
Parasitemia in mefloquine-treated, Plasmodium-infected volunteers (this study) or malaria patients. Black lines, treatment in this study with mefloquine; blue lines, treatment in earlier studies with mefloquine; red lines, treatment with chloroquine (CQ; 600/300 mg) (27, 28).

A readily apparent dose-response relationship was observed at the three dose levels of mefloquine (Fig. 3). At the lowest dose, 5 mg/kg, the drug stalled parasite growth but did not effect clearance, and rescue treatment with artemether-lumefantrine was required after 60 h because subjects began to become symptomatic. At the intermediate dose of 10 mg/kg, after an initial lag time of approximately 24 to 36 h (during which a surge in parasitemia was observed), a transient reduction in parasitemia was observed until approximately 96 h, when parasite growth began again and rescue treatment with artemether-lumefantrine was required. The 15-mg/kg dose of mefloquine was administered as a split dose of 10 mg/kg followed 4 h later by 5 mg/kg in an effort to reduce side effects. Given mefloquine's quick absorption and long half-life (Fig. 2), this split dose does not affect further PK/PD modeling. At this dose level, the lag time (before parasitemia begins to drop), as judged from visual inspection of the data, was shorter (approximately 12 to 24 h). This was followed by clearance of parasitemia, with no recrudescence observed.

Rates of clearance of parasitemia, as measured by the dose-specific PRR_max_ and log_10_ PRR_max_, were 157 (95% confidence interval [CI], 130 to 189) and 2.20 (95% CI, 2.11 to 2.28) for the 10-mg cohort and 195 (95% CI, 155 to 246) and 2.29 (95% CI, 2.19 to 2.39) for the 15-mg cohort. For the 10-mg/kg administration, the MIC of mefloquine was reached between 96 and 108 h. The geometric mean MIC was 784 ng/ml (CV = 18%). This is equivalent to an IC_50_ of 667 ng/ml, or 1.8 μM, comparable to the value we found in mice (768 ng/ml, or 2 μM).

### PD of mefloquine in published clinical trial data.

We identified two earlier clinical trial studies in which serial quantitative parasite count data were reported following administration of mefloquine monotherapy. Using the published data (Fig. 3), we calculated the PRR. In the first study, mefloquine was administered as a single oral dose of 500, 750, or 1,000 mg in a phase II trial undertaken with 147 adult male patients with acute, uncomplicated falciparum malaria admitted to the Hospital for Tropical Diseases, Bangkok, Thailand (27). In the second, 50 male Zambian patients with symptomatic falciparum malaria were treated with a single 1,000-mg dose of mefloquine (28) (and chloroquine [Fig. 3]). In the first study, at doses of 500 mg, 750 mg, and 1,000 mg, the respective PRRs were 802 (95% CI, 76 to 8,505), 1,031 (95% CI, 195 to 5,458), and 5,610 (95% CI, 119 to 265,238). In the second study, in which 1,000 mg of mefloquine was administered, the PRR was calculated as 77.5 (95% CI, 27 to 224).

### Correlating the SCID/Hu mouse and the human challenge models.

For mefloquine, blood exposure can be compared directly between humans and rodents because levels of plasma (protein) binding are roughly equivalent, measured at 95 to 96% in humans and ∼97% in rodents (29). The estimate of IC_50_ derived in NSG mice infected with P. falciparum, 768 ng/ml, closely matches that estimated by modeling in infected humans (665 ng/ml [23]).

## DISCUSSION

We found that mefloquine-induced parasite clearance kinetics observed in the IBSM matched those reported in earlier clinical studies (27, 28), despite an approximately 10,000-fold difference in parasite load. Further, comparison of clearance kinetics between the volunteers in our study and malaria patients in the two previously published studies showed that the reduction in parasitemia followed similar gradients for similar doses of mefloquine, with the same lag time (Fig. 3). This delay in onset of activity cannot be explained by a delay in reaching an exposure sufficient to exert a parasiticidal effect (Fig. 1A), suggesting that this lag is mechanism related. Although the antimalarial mode of action of mefloquine is not well understood, it has been proposed to be mediated by inhibition of heme polymerization. Thus, the lag may reflect the time required for nonpolymerized heme to accumulate to toxic levels, or for this toxicity to exert its effect. Alternatively, mefloquine could affect parasites only at a specific stage of their asexual reproduction cycle. The fact that we saw a similar lag for synchronized parasites in the challenge model and in clinical patients suggests that it is the time required for the toxic free heme to accumulate and exert its toxic effect rather than a life cycle-stage-specific effect.

Mefloquine exposure in volunteers (Fig. 1A) was consistent with earlier findings (19). The key parameters from this PK model and observed data correspond well with those reported previously for mefloquine in different human settings (30). Overall, a good correspondence was observed for the *in vivo* IC_50_ measured in the mouse and human challenge models and in clinical patients. However, PRRs differ substantially between the models and also between published clinical studies, suggesting that the IC_50_s may be more useful in predicting optimal dosing of new drugs. Moreover, other factors, including parasite biology and immunity (31, 32), have a significant effect on rates of clearance. From this perspective, the performance of antimalarials in the IBSM model offers some advantages, including the facts that immunity cannot confound drug clearance and that a range of candidate antimalarials can be compared under similar circumstances.

*In vitro* evaluation of drug efficacy in P. falciparum strains is relatively straightforward. However, an understanding of the *in vivo* interplay between a drug's changing exposure over time and the associated parasite growth/killing dynamics is critical in designing clinical studies needed for antimalarial drug development. Selection of an inappropriate dose in phase II or phase III studies may significantly prolong development or, worse, result in discontinuation of development of a drug with good potential. Antimalarials, especially those providing fast clearance of blood-stage parasites (Medicines for Malaria Venture target candidate profile 1 [1, 33]), are intended for use in large numbers of patients, many of whom may be vulnerable due to additional disease, malnutrition, young age, and/or variable immune status. This makes it even more important to optimize safe and effective dosing.

While falciparum malaria models involving both NSG mice and human volunteers have been developed and refined recently, a gap to date has been a formal assessment of these models' validity in a coherent pathway for drug discovery using a clinically well-established antimalarial as a benchmark. In this study, we have evaluated mefloquine both in infected NSG mice and in volunteers, fitted the key parameters in a PD model, and found an excellent correlation between the variables that describe each *in vivo* system. It remains to be seen if the correlations that we have found between the *in vivo* model and in volunteers also hold for drugs with a different mechanism of action, with exposure characteristics different from those of mefloquine, and that produce significantly different exposures in rodents and humans. But with antimalarials typically targeting either the parasite directly or components in the human infected erythrocyte compartment, it would seem that the two models that we have evaluated in this study have all the critical elements in place for confidently guiding clinical development of molecules currently progressing through the antimalarial pipeline.

## Supplementary Material

Supplemental material
