# Supplementary material for: Linking Murine and Human Plasmodium falciparum Challenge Models in a Translational Path for Antimalarial Drug Development
Source: Antimicrob Agents Chemother. 2016 May 23;60(6):3669–75. doi: 10.1128/AAC.02883-15 (PMC4879391; doi:10.1128/AAC.02883-15)
Supplement: Supplemental material [file supp_60_6_3669__index.html]

Linking Murine and Human Plasmodium falciparum Challenge Models in a Translational Path for Antimalarial Drug Development — Supplemental material 

# Linking Murine and Human Plasmodium falciparum Challenge Models in a Translational Path for Antimalarial Drug Development

## Supplemental material

- Supplemental file 1 -

  Supplemental text, Fig. S1-S4, and Tables S1 and S2

  PDF, 274K
