# Supplementary material for: Linking Murine and Human Plasmodium falciparum Challenge Models in a Translational Path for Antimalarial Drug Development
Source: Antimicrob Agents Chemother. 2016 May 23;60(6):3669–75. doi: 10.1128/AAC.02883-15 (PMC4879391; doi:10.1128/AAC.02883-15)
Supplement: Supplemental material [file AAC.02883-15_zac006165244so1.pdf]

Procedures

Modelling Methods

Nonlinear mixed effects modeling of data

Nonlinear mixed effects modeling was used to analyze the PK/PD data from the SCID model and the PK from the human challenge study.

Pharmacokinetic and PK/PD data were modeled separately using nonlinear mixed-effects modeling with NONMEM software (Version 7.2, ICON Development Solutions, MD). Post processing and automation was performed using software including the Pearl-Speaks-NONMEM programs in the programming language R (Version 2.12, The R Foundation for Statistical Computing). Preliminary modeling of the data to determine appropriate structural models and to investigate the data was performed using either first order or first order conditional estimation (FOCE) methods. The final model was estimated using the first-order conditional estimation method with interactions and results reported if convergence is possible. Model suitability and selection was assessed from the objective function values (OFV; computed by NONMEM as minus twice the log likelihood of the data), goodness-of-fit graphical analyses, and physiological plausibility. Different P values were used as statistical criteria at different stages during the model-building process, as described below (changes in OFV [ΔOFV] of 3.84, 6.63, and 10.8 are considered significant at $P < 0.05$, $P < 0.01$, and $P < 0.001$, respectively, with 1 degree of freedom).

There were no PK data that were below the limit of quantification. Pharmacodynamic data (parasitemia data) below the limit of detection was treated in the following manner: the first data point in the time course was set equal to the limit of quantification.
Inter-individual random variability in all parameters will be modeled exponentially:

$$\beta_i \beta_i = TV(\beta) \times \exp(\eta_{i,\beta})$$

Equation 1

where

- β_i is the individually estimated parameter value of β for the ith patient,
- TV(β) is the typical parameter value for the population, and
- $\eta_{i,\beta}$ is the inter-individual random variability for the described parameter.

The inter-individual random variability is assumed to be normally distributed with a zero mean and variance ω^2. The residual random variability was assumed to be multiplicative Confidence intervals for the final parameter estimates were calculated using the PsN bootstrap function. Goodness-of-fit characteristics were evaluated by plotting observed drug concentrations against population predicted and individually predicted drug concentrations and by plotting conditional weighted residuals against population predicted drug concentrations and time. Data checks and diagnostics including numerical and visual predicted checks and nonparametric bootstrap diagnostics were obtained. The final model with estimated variability was used to simulate 2,000 concentrations at each sampling time point (binning will be centered on protocol time points), and the 95% confidence intervals around the simulated 5th, 50th, and 95th percentiles were overlaid with the observed data to evaluate the predictive power of the model (visual predictive check).

Structural models for modeling SCID PK/PD data

Mouse Baseline PD

The PD response in the absence of mefloquine was measured in 6 SCID mice infected with *P. falciparum* and dosed *p.o.* with vehicle.

Visual inspection of the baseline parasitemia data showed it to be log-linear and so a mono-exponential function (Equation 2) was fitted to the data. The mono-exponential function was:

$$P(t)=P_{t=0}\times\exp(k_t\times TIME)$$

Equation 2

Where:

- P = parasitemia (at time = t [P(t)] or at start [$P_{t=0}$])
- k_t = first order parasite growth constant

This was transformed into its equivalent differential equation in anticipation of a more complex equation once drug effects were integrated:

$$\frac{dP}{dt}=k_G\times P(t)=Growth$$

Equation 3

The parasite growth described is the balance of parasite growth rate and their intrinsic death rate which is assumed to be constant over the density of parasites observed. This validity of this assumption may decrease as parasitemia reaches ceiling densities - no evidence of a ceiling density has been observed in the SCID model.

Fig. S1: Parasitaemia in *P. falciparum*-infected (non-treated) NOD mice (individual animals, semi-log scale).

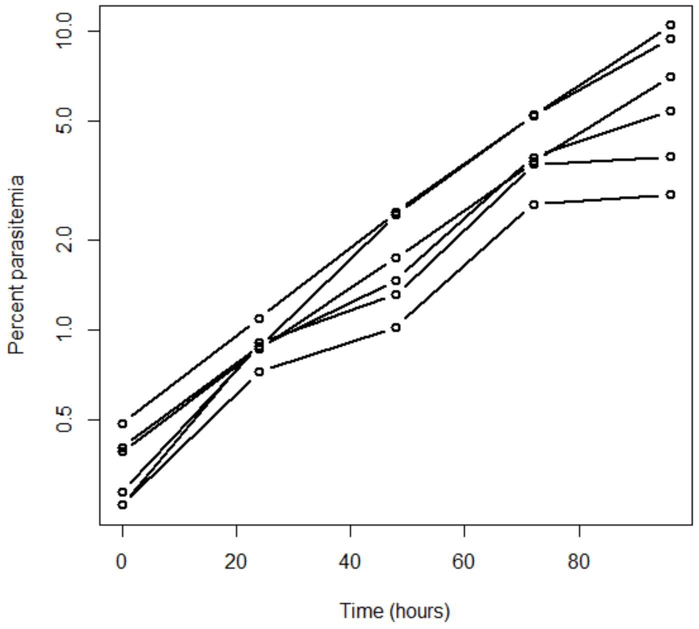

Fig. S2: Mefloquine exposure and efficacy in SCID mice. Ratios of predicted:observed data points *vs.* time overlaid with a spline through all data. Equivalent values have a ratio of 1 denoted by the horizontal blue line.

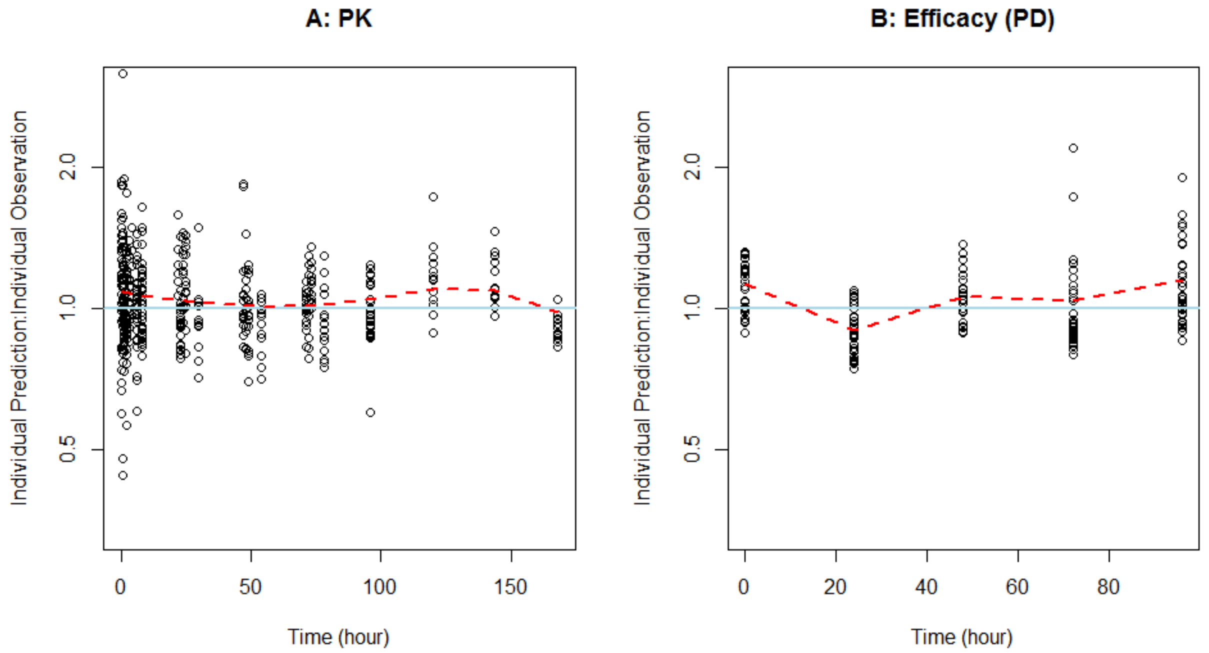

Fig. S3: Goodness of fit plots for parasitemia and PK modelling following simultaneous modelling with the PK/PD model.

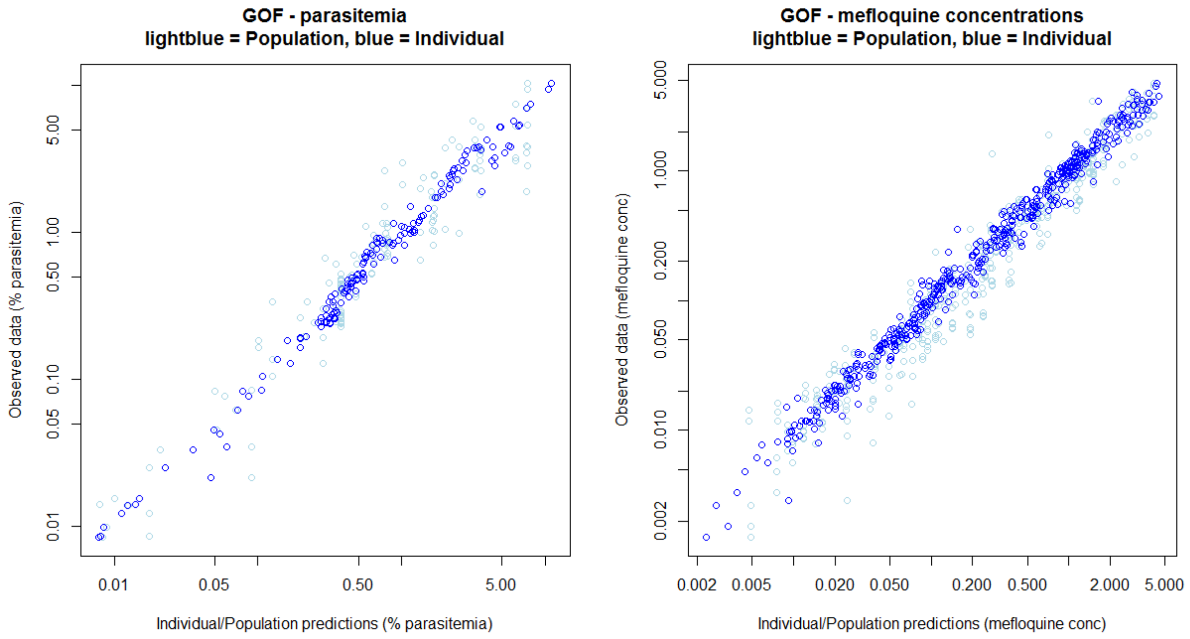

Fig. S4: The Effect of Mefloquine dose on AUC_{96} (left panel) and C_{max} (right panel).
Both axes are log-transformed to illustrate the linearity relationship: $\theta = a \times Dose^b$ where θ is the parameter considered. The exponent b (exponential gradient) is reported with its 2.5^{th} and 97.5^{th} confidence intervals. The exponential relationship was significant with the intercept and gradient being highly significant ($p<0.0001$).

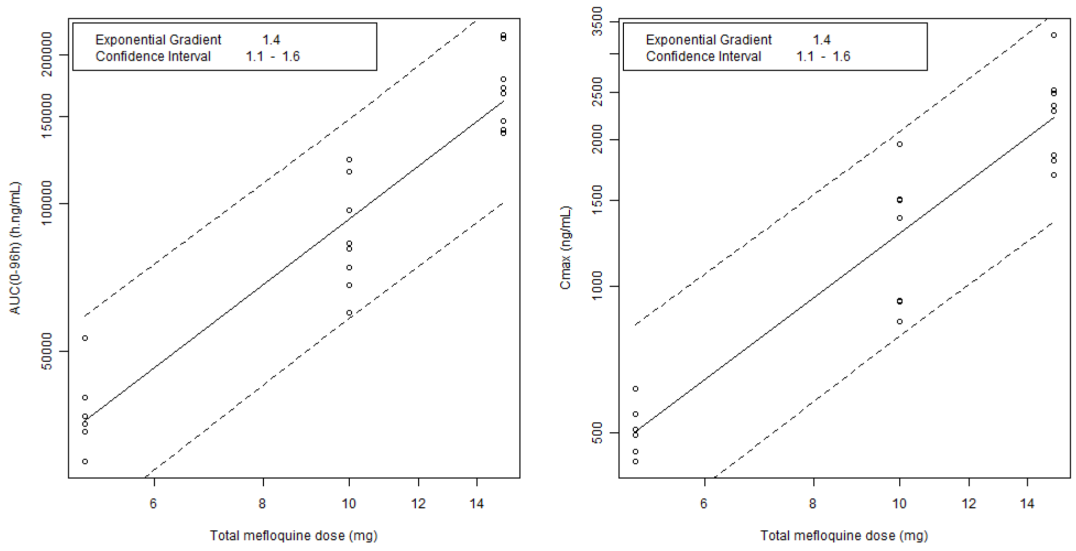

Table S1 Non-compartmental PK parameters (with 95% confidence interval) derived from mefloquine-treated subjects in the human challenge model

Dose (mg)	C_{max} (μg /ml)	T_{max} (h)	AUC_{96} (h.μg/ml)
**5**	0.51<br>(0.44 – 0.62)	4<br>(3 - 96)	37.8 (30 - 53)
**10**	1.3<br>(0.85 – 2.0)	4<br>(3 - 48)	123(88 - 175)
**15**	2.2<br>(1.7 – 3.3)	24<br>(7 - 72)	240 (200 - 303)

Table S2 Overview of adverse events

The Total Number of Participants with AEs	22
The Total Number of AEs	150
The Total Number of AEs that were probably related to study drug	74
The Total Number of AEs that were possibly related to study drug	41
The Total Number of AEs that were unlikely related to study drug	17
The Total Number of AEs that were unrelated to the study drug	18
The Total Number of AEs with a severity of severe	3
The Total Number of AEs that were serious	1
The Total Number of AEs that were not resolved at the end of the study	1

References

1. Rockett RJ, Tozer SJ, Peatey C, et al. A real-time, quantitative PCR method using hydrolysis probes for the monitoring of Plasmodium falciparum load in experimentally infected human volunteers. Malar J 2011; 10:48.
2. Angulo-Barturen I, Jimenez-Diaz MB, Mulet T, et al. A murine model of falciparum-malaria by in vivo selection of competent strains in non-myelodepleted mice engrafted with human erythrocytes. PLoS One 2008; 3:e2252.
